# A Sketch of the Life and Character of Dr. Thomas Hinde

**Published:** 1829-03

**Authors:** 


ART. IV.—*A Sketch of the Life & Character of Dr. Thomas Hinde.* By the EDITOR.

In the 5th number of this volume we took occasion to notice the age and character of the oldest patriarch of the profession in America, Dr. Holyoke, of Salem, Massachusetts. In the present number we propose to say something of one of his cotemporaries, who lately descended into the "valley and shadow of death."

But few of the physicians of Cincinnati are aware, that one of the most ancient, and in his day successful, members of the profession, has resided for the last 18 or 20 years and died within a few months, in their immediate neighbourhood. Dr. THOMAS HINDE, to whom we refer, expired in the autumn of 1828 at Newport, Kentucky, at the venerable age of 92 years. Commencing the practice of his profession at an early and continuing it to an advanced age, few men of any country were ever devoted to its arduous and responsible duties for a longer period. Yet he has, like the majority of the profession, left behind him no written memorial, of his unwearied labours in the great cause of humanity. When a humane and skilful physician expires before he has passed the scriptural limit of human life, scarcely any event is seen to agitate society with a gloomier feeling of sorrow. A long train of mourning friends and patients, attest how deeply he had inscribed himself on the hearts of all around him. Indeed, we have long thought, that the honours thus awarded, might fill the measure of any man's ambition. It was, however, the fate of Dr. H. to survive the majority of those to whom he had administered health and comfort; to outlive his own well earned reputation; to see not merely the beginning, increase and meridian, but the decline and extinction of his professional fame, perisning, as it necessarily did, with the beneficiaries of his skill and kindness.

To those who thirst after posthumous distinction, and would leave a name to be named with reverence by the generations that are to follow them, the life of Dr. H. may afford a salutary lesson. To affectuate their aim, they *must write*, as well as observe and think. If they do not record their experience in the annals of the profession, 'their works will follow them.' In Medicine, none should hope to live with the ages which are to come, who do not write. But a different and nobler reason may be urged for this duty. The improvements which a physician makes, are lost to the profession, unless committed to the press; and thus the benefits which he might confer on future times never reach them. If all the practical discoveries which have been made since the revival of Medicine in Europe, and never communicated to the public, could now be recovered and embodied, it is an allowable presumption, that they would constitute a most valuable addition to the maxims and formulæ of the Profession. Every one must recollect, to have seen physicians of age, extensive experience, and acute observation, descend to the grave without having contributed their due proportion, or indeed, any proportion at all, to the stock of facts which it is the duty of all to augment. We remember several practioners of this class, who like Dr. H. left no written memorials of their success; while, like himself, they might, we believe, have contributed something of permanent utility to the science. Several causes might be assigned for this omission; and although it may be considered out of place, we shall venture to name one of them. In the minds of many physicians, there is a wrong estimate of the contributions which certain writers have made to the profession. They are valued too high; an error which would demand no correction, if it did not discourage those who can contribute but a mite, from casting that mite into common treasury. The truth is, that the longest writers have not been the greatest contributors; and that the amount of *new* facts with which the most voluminous authors have enriched our archives, is much less than many suppose, and ought not to deter any one from publishing the smallest discovery which he knows to be real. But we must return to the subject of our sketch.

Dr. H. was born in Oxfordshire, England, on the 10th of July, 1737. After receiving a suitable degree of classical education he was sent to London, to study physic and surgery. His principal tutor was Dr. Thomas Brooke, one of the physicians of St. Thomas' Hospital. The practice of this physician was embodied and published, by his brother, Dr. Robert Brooke, in two volumes, which we recollect to have read; though nearly half a century has elapsed since they were ordinary books of reference.

In the year 1757, at the early age of twenty, Mr. H. had made such proficiency, that his master presented him to the Royal College of Surgeons for a license. Sustaining a satisfactory examination, he immediately afterwards received the commission of Surgeon's mate in the navy; and sailed for America, with the forces under the command of Gen. Amherst.

He landed at New York on the 10th of June, 1757; and was afterwards, during the same year, with the squadron at Louisburg. The following winter he spent at Halifax; and in 1758, assisted in the reduction of Louisburg by Amherst. A new conquest was now meditated and our young Surgeon proceeded with the celebrated Gen. Wolfe, in his memorable expedition against Quebec. It was his good fortune to be attached to the ship which bore the commander in chief, where he had ample opportunities of seeing much of that distinguished man, and observing his operations. His reminiscences of these events, were among the most cherished of his life. Down to the day of his death he was accustomed to describe the General as "a tall and robust person with fair complexion and sandy hair; possessing a countenance calm, resolute, confident, and beaming with intelligence."

Dr. H. was near the General at the moment of his fall, and when an aid exclaimed, 'they run, they run,' the Doctor heard the expiring chief articulate the question, 'who run?' He was answered, 'the French, sir; they are running away in all directions:' 'then,' said he, 'I die contented,' and sinking into the arms of the officer who supported him, he expired.

This celebrated death scene has been often painted, and in some of the pictures Dr. Hinde is represented as being present, and feeling the pulse of the wounded General.

After this event Dr. Hinde was present at the reduction of Bell'isle. Having experienced promotion, he remained in the service as surgeon of a vessel of war, after the peace of 1763; but was at length induced to abandon the navy for a residence in the colonies. An aged physician of Virginia had written to Dr. Brooke, requesting him to send out from London, a young physician of talents, who might assist him in business. Dr. Brooke selected for this purpose his pupil Dr. Hinde, and earnestly advised him to avail himself of the situation which was offered. To this influence was added that of a kinsman in Virginia, and a gentleman in the same state, who had been his friend and fellow student in London. Yielding to their united importunities, he resigned his commission and set sail for the state just mentioned, where in 1765 he settled as a physician. His first residence was in Essex county; but he soon afterwards left it for King and Queen, where he purchased property and engaged in the practice of medicine and surgery with great success. In 1767 he married Miss Mary Todd Hubbard, daughter of his countryman Mr. Benjaman Hubbard, an English merchant; by whom he had eight children, and with whom he lived 61 years. For reasons which are unknown to us Dr. Hinde did not continue long in this situation, and his next removal was to Hanover county, where he established himself in the neighborhood of the celebrated orator and statesman, Patrick Henry, and became his family physician. He was also in the vicinity of another eminent man, the Reverend Samuel Davis, afterwards President of Princeton college. To the society of these great men, especially the former, the Doctor was chiefly indebted for his conversion from Royalty to Republicanism. Under the powerful influence of the orator and patriot, the Doctor forgot both his king and native country, and engaged with zeal and activity in the eventful cause of the oppressed colonies. Lord Dunmore, the Governor of Virginia, was his countryman and personal friend, and they had done each other acts of kindness; but in the mind of the Doctor, all personal considerations were absorbed by those involved in the approaching contest; and he became Henry's chief Surgeon in 1775, when he marched against Lord Dunmore, on what was called the gun powder expedition. Subsequently he prepared himself to act as Surgeon to the regiment, which Henry was destined to command; but relinquished the design, when a new direction was given to the efforts of his friend, by his being elected Governor of Virginia. The Doctor, however, did not become an idler in the cause of liberty; possessing the confidence of the Governor, he was appointed to inoculate the troops designed to enter the continental service. To this responsible duty, he devoted himself with a zeal which conferred on his adopted country the most important benefits, while it seriously impaired his private fortune.

After the termination of the war, he continued for many years to reside in Hanover county, dispensing the benefits of his profession with distinguished effect.

Dr. Hinde had been educated in the principles and practice of the Episcopal Church; but he had now become a Deist, and took pride and pleasure in ridiculing Christianity. His views and feelings, however, at length underwent a radical change; the immediate cause of which was so uncommon, and, at the same time, so professional, that we shall not hesitate to relate it. His wife and daughter had been converted to Christianity, and attached themselves to the Methodist Episcopal Church. For this act, his daughter was banished from his house, and his wife placed under medical treatment, for what he considered, or affected to consider, insanity. His remedy was a blistering plaster to the whole length of the spine, which he left on for several days. By this measure of violence, he hoped to deter her from further attendance on places of public worship. But, as he used to say, God turned the 'huge blister' upon his own heart. The christian fortitude and meekness, with which his wife bore the protracted anguish which his cruelty inflicted on her, excited his sympathy and filled his soul with remorse. A feeling of respect was awakened towards that religion, whose votaries could endure such pain and persecution, without a murmur; and he was led forthwith to investigate its origin and principles. The inquiry resulted in a perfect conviction of its divinity; and he attached himself to the same church, from which he had sought by violence to estrange his wife and daughter; and for nearly half a century continued one of its most devout and exemplary members. Such was his temperament, indeed, that he may fairly be said to have passed into the opposite extreme. Down to his dying day, religion was his darling theme. No waking hour ever passed, whoever might be present, in which he did not utter some expression of admiration for the christian faith, and thank heaven that he felt its influence. Throughout the whole of this long period, he never attended to the call of a patient, without first retiring to pray in secret, for the success of what he might prescribe; and when he reached the house, whether of rich or poor, christian or infidel, his invariable practice was, to assemble such members of the family as could be conveniently brought together, and re-engage, with them, in prayer for the recovery of the sick, before he would exhibit a single remedy. In the efficacy of prayer the Doctor was a firm believer, and not a few of his patients cherished the same faith. To all such, his religious efforts were auxiliary to his professional; as they contributed to tranquilize the feelings, inspirit the hopes, or confirm the resignation of the sick. Indeed it has long appeared to us, that religion is in some degree a natural inmate of the sick chamber. Most people, when seriously ill, become affected either with religious feelings, or superstitious horrors; which may frequently be quieted, or made to take a most useful direction, by the presence and conversation of judicious christians. However this may be, I am not aware that Dr. Hinde's patients, at any time, suffered injury from his persevering discharge of what he undoubtedly regarded as a solemn duty.

In 1797, Dr. H. left Hanover for Kentucky, and fixed his residence in Clark county, where he speedily acquired an extensive practice, especially in Surgery. This was the last theatre of his professional exertions. The long but bright evening of his life, was spent in Newport, where beloved and honoured he expired inthe midst of his descendants.

As a surgeon and physician, Dr. H. for a period much longer than most men follow the profession, devoted himself to its duties, in a manner and with effects, which inspired universal confidence. He performed most of the higher operations in surgery, for which his service in the navy nearly eight years, had peculiarly qualified him. In military surgery his practice had, indeed, been great; as he was attached to the army and navy during a period in which many desperate battles were fought. After some of these, he amputed limbs, to use his own familiar language, 'by the cart-load.' Nevertheless, he does not seem to have been prone to resort to the knife; but, in general, to have regarded it as the *ultima ratio artis*. His great object was to render it unnecessary, by an appropriate plan of treatment; and this object, from his diversified experience and great natural sagacity, he frequently accomplished under circumstances so discouraging, that other practitioners had lost hope in every thing but the scalpel. On this subject his example is worthy of all imitation.

In physic Dr. H. was not less successful than in surgery. He was practically acquainted with the diseases of ships and armies; and during his long residence in Hanover county, at that time, annually infested with autumnal fever, he acquired great skill and character in the cure of that epidemic. In the treatment of small-pox, from the duty assigned to him by Governor Henry, he had a degree of experience which has seldom fallen to the lot of any individual. At one time, with the aid of a single assistant, he had 1500 patients under his care, with that malady.

Dr. Hinde was an acute physiogomist. He had studied diligently the rules of Lavater, correcting and augmenting them by his own observations. He frequently averred, what no well informed person will venture to deny, that his knowledge of physiognomy was of great use to him in the practice of his profession. By long and attentive observations on the human countenance, he came to know by a *coup d'œil*, not only much of a patients thoughts and feelings, but also a great deal relative to his temperament and diseases. On this point not less than the last, his example deserves to be followed; forevery physician should cultivate his observing faculties, and nothing in the aspect of the sick should ever escape him.

The incessant exercise of this faculty, led the Doctor to observe many things connected with the constitutions and health of his friends and acquaintances. He was, indeed, in the habit of studying their physical, not less than their intellectual character, and accustomed, at all times, to express his convictions freely. He thus, in many instances, warned them in time of approaching danger; indicated to individuals the diseases to which they were constitutionally liable; and thereby directed their attention to the appropriate means of prevention,—a superintendence which every practioner should exercise over those who call him their 'family physician.'

His admonitions and prophecies should, however, be delivered prudently, or they may do harm instead of good. In the following case it is not altogether clear, that the prediction did not contribute to its own verification.

'Mr. Ellis,' said the Doctor to one of his Virginia friends, 'take care! I know your constitutional tendencies; you are in danger of speedy death.' 'Doctor,' he replied, 'I never was so hearty in my life.' 'For that very reason,' rejoined the Doctor, 'you are in great danger. From your form and temperament, you will probably go off in an apoplectic fit. Life ebbs and flows; you are at high tide; your system must sustain a shock!' In about two weeks Mr. Ellis was no more; but the gentleman who heard the dialogue, does not state of what disease the patient died.

In his attention to the sick, in the active stages of his life, Dr. H. was unremitting and exemplary. When the infirmities of age began to gather upon him, like all others he felt the labours of a country practice irksome; still, however, he was capable of being aroused to exertion by whatever strongly addressed itself to his *sense of duty as a christian.* The following anedote, often related by himself, may serve to illustrate his character on this point.

When residing, at an advanced age, in the interior of Kentucky, he had a visit of several days from his old and excellent friend Bishop Asbury. During the time, he was sent for to visit a patient at some distance, who was alarmingly ill. The night was dark and rainy, and the Doctor, much afflicted with vertigo, hesitated to obey the summons. The messenger was greatly distressed; when the Bishop, who had been awakened and overheard the conversation, exclaimed—'Doctor you *must* go!' 'Good Lord,' he used afterwards to say, 'the words appeared to me, as though they came from Heaven! I went and found the patient in agony—a few hours more and he would have been past recovery—his case was desperate indeed! I first prayed and committed him to God; then applied medical aid and used every possible means till morning, when, to the great joy of his family and friends, he was entirely relieved, and offered up thanks to God. I have always regarded this event as a singular providence.'

After what has just been said, it is almost superfluous to add, that Dr. H. was a consistent and untiring attendant on public worship. His taste and judgement kept him in communion with the respectable sect of christians, to which he, at first, attached himself; but he cherished the kindest feelings towards all others, of which he gave many practical evidences. The zeal which he felt when united with his brethren in devotional exercises, was, in the language of chemistry, highly *effervescent;* and frequently manifested itself in *ebullitions* of feeling, the most artless and unaffected. Instances of this kind have been mentioned to us; one of which, as illustrating this feature of his character, we shall present to our readers. The Doctor was in the habit of indulging himself in the extempore responses, which are practised by the devouter members of the methodist church while the clergyman is preaching. In his old age, he frequently indulged himself in these pious exclamations in the argumentative periods of the discourse, when others were not so strongly excited, and wished to attend, without interruption, to the reasonings of the preacher. Some of his friends admonished him on this subject, and he promised in future to restrain himself. But, as might have been foreseen, he became impatient under this rash acquiescence; and not long afterwards, in the midst of a sermon, becoming warmed in advance of the congregation but still recollecting his promise, with a *naivete* which might challenge comparison, he exclaimed—'*Amen, at a venture!*'

Fervent and uncompromising as was the devotional spirit of the deceased, it gave birth to no austerity. His was the religion of love, which delighted in the cheerfulness and innocent enjoyment of all around him. If an enthusiast, he was not an anchorite; he neither shunned nor repelled society; and few men ever attained in a higher degree, to the pleasant and benevolent manners, which should peculiarly characterize a physician and a christian. Being an acute observer, and blessed with a retentive memory, his mind was stored with interesting anecdotes and narratives, relating to distinguished persons and remarkable events; and the ease and simplicity with which they came forth in conversation, rendered an intercourse with him both instructive and entertaining. His colloquial powers were, indeed, of an original cast; & their effect was heightened by the mild but searching expresssion of his light blue eyes. Even in the last year of his pilgrimage, their fires were not quenched; and a gentleman, after being introduced to him, observed: 'he seemed to ask with his eyes, are you an honest man?'

As every physician ought, Dr. Hinde cultivated his domestic relations, and drew from this source copious draughts of happiness. To his children, grand children, and great grand children, he was a kind and loving father; to his wife, a lady of strong and cultivated intellect, who still lives though nearly of his own age, he was a most affectionate husband; and the only regret he expressed on the approach of death was, that he should leave behind him the companion of sixty years. An interesting display of conjugal feeling for a *nonagenarian*.

In concluding our sketch, we may be allowed to express a regret, that one endowed with so much sound understanding, quick perception, and active benevolence; favoured with such diversified opportunities, and permitted to live so long, should have left behind him none of the fruits of his ample experience. When such a man bequeaths to posterity nothing but his good name, he can scarely be said to have fulfilled his destiny.

[Compiled *chiefly* from MSS. furnished by the family.—Ed.]

